# Supplementary material for: A generic model of life satisfaction: The case study of parkrun
Source: PLOS Glob Public Health. 2025 Oct 2;5(10):e0005065. doi: 10.1371/journal.pgph.0005065 (PMC12490765; doi:10.1371/journal.pgph.0005065)
Supplement: S1 Text — (DOCX) [file pgph.0005065.s005.docx]

# File S1 Text: 2024 UK survey of parkrun.

**parkrun health and wellbeing survey**

In this survey, parkrun and Sheffield Hallam University are asking about your health and wellbeing to better understand the impact of taking part in parkrun events.

This survey will take around 10 minutes to complete, but take as much time as you need. You can choose not to answer some questions by moving onto the next question. Compulsory questions are marked with an asterisk *.

Taking part in this survey is your choice. If you decide to take part, you may leave the survey at any time, but your answers already provided will be saved and used in the analysis. By completing this survey, you agree for us to access your parkrun participation data. Your personal data will be handled at all times in accordance with parkrun's [privacy policy](https://www.parkrun.com/privacy/). All your parkrun data and answers to this survey will be pseudonymised (pseudonymisation is a technique that replaces or removes information in a data set that identifies an individual).

Please click [here](https://shusls.eu.qualtrics.com/CP/File.php?F=F_es2YepEiN5dM9GC) to read the full participant information sheet.

For questions or concerns about this survey or how we will use your data, please contact the research team: [parkrunsurveys@shu.ac.uk](mailto:parkrunsurveys@shu.ac.uk)
If you have a general parkrun query, please contact parkrun [here](https://support.parkrun.com/hc/en-us/requests/new).

Please note, this survey is currently within the pilot phase of the study. Your responses and data will be used to test the questionnaire. Because of this, it is possible that your data will not be used in the final analysis of the study.

**Giving consent to take part in this survey**

I have read (or someone has read to me) this information and the participant information sheet, and I understand that I am being asked to complete a survey about my health and wellbeing.

Once data collection closes, parkrun will receive the SHA-2 code embedded into my survey link and extract my parkrun participation and registration details from their database. These will be shared with the research team and matched to my survey responses. I understand that none of my data will be used or shared in a way that can identify me personally. By completing this survey, I consent that my parkrun details will be added to my survey responses in this way.

I agree for my pseudonymised parkrun data to be used for research purposes.

- I consent to participate in this study
- I DO NOT consent to participate in this study

Please specify your date of birth:

- Day (0-31 drop down menu)
- Month (January to December drop down menu)
- Year (1910 to 2008 drop down menu)

Please specify your gender:

- Male
- Female
- Another gender identity
- Prefer not to say

Over the last 4 weeks, how often have you done at least 30 minutes of moderate exercise (enough to raise your breathing rate)?

- Less than once per week
- About once per week
- About twice per week
- About three times per week
- Four or more times per week
- Rather not say/ don’t know

Choose one option that best describes your participation at parkrun:

- Runner or walker only
- Volunteer only
- Runner or walker and volunteer

What is your ethnic group?

- White – English/Welsh/Scottish/Northern Irish/British
- White – Irish
- White – Gypsy or Irish Traveller
- White – Any other White background (please describe in the next question)
- Mixed/Multiple ethnic groups – White and Black Caribbean
- Mixed/Multiple ethnic groups – White and Black African
- Mixed/Multiple ethnic groups – White and Asian
- Mixed/Multiple ethnic groups – Any other Mixed/Multiple ethnic background (please describe in the next question)
- Asian/British Asian – Indian
- Asian/British Asian – Pakistani
- Asian/British Asian – Bangladeshi
- Asian/British Asian – Chinese
- Asian/British Asian – Any other Asian/British Asian background (please describe in the next question)
- Other ethnic group – Arab
- Other ethnic group – Any other ethnic group (please describe in the next question)
- Prefer not to say
- If selected White – Any other background (please describe in the next question)

If you selected “White – Any other White Background” please describe it here:

[empty text box]

- If selected Mixed/Multiple ethnic groups – Any other Mixed/Multiple ethnic background (please describe in the next question)

If you selected “Mixed/Multiple ethnic groups – Any other Mixed/Multiple ethnic background” please describe it here:

[empty text box]

- If selected Black/African/Caribbean/Black British – Any other Black/African/Caribbean/Black British background (please describe in the next question)

If you selected "Black/African/Caribbean/Black British - Any other Black/African/Caribbean/Black British background" please describe it here:

[empty text box]

- If selected Other ethnic group – Any other ethnic group (please describe in the next question)

If you selected “Any other ethnic group” please describe it here:

[empty text box]

We are interested in the leisure time physical activity parkrunners do as part of their everyday lives.

In the past week, on how many days have you done a total of 30 minutes or more of physical activity, which was enough to raise your breathing rate?
This may include sport, exercise, and brisk walking or cycling or to get to and from places, but should not include housework or physical activity that may be part of your job.

- 0 days
- 1 day
- 2 days
- 3 days
- 4 days
- 5 days
- 6 days
- 7 days

How is your general health?

- Very good
- Good
- Fair
- Bad
- Very bad

Do you have any physical or mental health conditions or illnesses or lasting or expected to last 12 months or more?

- Yes
- No
- If Yes is selected for Do you have any physical or mental conditions or illnesses lasting or expected to last 12 months or more?

Do any of your health conditions or illnesses reduce your ability to carry out day-to-day activities?

- Yes, a lot
- Yes, a little
- Not at all
- If Yes is selected for Do you have any physical or mental conditions or illnesses lasting or expected to last 12 months or more?

What is your health condition or illness?
Please tick all that apply. If you select “cancer” or “other” you are able to specify in the next question.

- ADHD – Attention Deficit Hyperactivity Disorder
- Alcohol or Drug Addiction
- Allergies (any that limit your day-to-day activity)
- Alzheimer’s or dementia
- Anxiety Disorder
- Arrhythmia (abnormal heart rate) or Atrial Fibrillation (irregular heart rate)
- Arthritis
- Asthma
- Autism Spectrum Disorder
- Bipolar Disorder
- Cancer
- Carpal Tunnel Syndrome
- Chronic Migraines
- Chronic Pain
- COPD (chronic obstructive pulmonary disease) and Emphysema
- Coronary Artery Disease (including angina, peripheral vascular disease)
- Crohn’s Disease
- Degenerative Disc Disease
- Depression
- Disorders of the Spine
- Epilepsy or seizure disorder
- Endometriosis/Adenomyosis
- Fibromyalgia
- Gout
- Hearing loss or impairment
- Heart Failure
- Hepatitis
- High Blood Pressure (Hypertension)
- Interstitial Cystitis
- Irritable Bowel Syndrome
- Kidney failure or Chronic kidney disease
- Learning disability (e.g., dyslexia, dyspraxia)
- Liver Disease
- Long COVID
- Lupus, or Systemic Lupus Erythematosus (SLE)
- Lyme Disease
- Multiple Sclerosis (MS)
- Neuropathy, Peripheral Neuropathy
- Obesity
- Organic Mental Disorders (including Organic Brain Syndrome)
- Osteoporosis
- Panic Attacks
- Parkinson’s Disease
- Polycystic ovary syndrome (PCOS)
- Post Traumatic Stress Disorder (PTSD)
- Reflex Sympathetic Dystrophy (RSD)
- Rheumatoid Arthritis
- Ruptured Disc
- Schizophrenia
- Scoliosis
- Sleep Apnoea
- Stroke (TIA, CVA: Cerebrovascular Accident)
- Thyroid gland disorder
- Traumatic Brain Injury (TBI)
- Type 1 Diabetes
- Type 2 Diabetes
- Ulcerative Colitis
- Venous Thromboembolism (DVT; Deep Venous Thrombosis & Pulmonary Embolism)
- Vision loss or impairment
- Other
- Prefer not to say
- If What is your health condition of illness Cancer is selected

If you ticked “cancer”, please specify:

- Bladder
- Bowel
- Brain, other CNS and intracranial Tumors
- Breast
- Cancer of unknown primary
- Head and Neck
- Kidney
- Leukaemia
- Liver
- Lung
- Melanoma Skin Cancer
- Myeloma
- Non-Hodgkin Lymphoma
- Oesophagus
- Ovary
- Pancreas
- Prostate
- Stomach
- Thyroid
- Uterus
- Another type of cancer – please specify [empty text box]
- If other was selected in What is your health condition or illness

If you ticked “other”, please specify your condition or illness:

[empty text box]

Overall, how satisfied are you with your life nowadays?
Where 0 is ‘not at all’ and 10 is ‘completely’.
[drop down menu 0 to 10]

Overall, how happy did you feel yesterday?
Where 0 is ‘not at all’ and 10 is ‘completely’.
[drop down menu 0 to 10]

On a scale where 0 is ‘not at all anxious’ and 10 is ‘completely anxious’, overall how anxious did you feel yesterday?
[drop down menu 0 to 10]

Overall, to what extent do you feel that the things you do in your life are worthwhile?
where 0 is ‘not at all’ and 10 is ‘completely’.
[drop down menu 0 to 10]

- If participants chose Runner or walker only or Runner or walker and volunteer

I first participated in parkrun as a runner or walker

|  | Not at all true 1 | 2 | 3 | 4 | Very true for me 5 |
| --- | --- | --- | --- | --- | --- |
| To contribute to my physical health |  |  |  |  |  |
| To contribute to my mental wellbeing |  |  |  |  |  |
| To contribute to my life satisfaction |  |  |  |  |  |
| To gain a sense of personal achievement |  |  |  |  |  |
| To contribute to my happiness |  |  |  |  |  |
| To be active in a non-judgemental environment |  |  |  |  |  |
| To be active in a physically safe environment |  |  |  |  |  |
| To contribute to my fitness |  |  |  |  |  |
| To spend with my family, friends or colleagues |  |  |  |  |  |
| To encourage or support someone else (e.g. family, friends or colleagues) |  |  |  |  |  |
| To feel part of a community |  |  |  |  |  |
| To manage my weight |  |  |  |  |  |
| To improve my confidence |  |  |  |  |  |
| To compete against others |  |  |  |  |  |
| To compete against myself |  |  |  |  |  |
| To be active |  |  |  |  |  |
| To be outdoors |  |  |  |  |  |
| To meet new people |  |  |  |  |  |
| To have fun |  |  |  |  |  |
| To manage my health condition, disability or illness |  |  |  |  |  |
| Other [please specify] |  |  |  |  |  |

- If participants chose Volunteer only or Runner or walker and volunteer

I first volunteered at parkrun…

|  | Not at all true 1 | 2 | 3 | 4 | Very true for me 5 |
| --- | --- | --- | --- | --- | --- |
| To contribute to my physical health |  |  |  |  |  |
| To contribute to my mental wellbeing |  |  |  |  |  |
| To contribute to my life satisfaction |  |  |  |  |  |
| To gain a sense of personal achievement |  |  |  |  |  |
| To contribute to my happiness |  |  |  |  |  |
| To feel part of a community |  |  |  |  |  |
| To spend with my family, friends or colleagues |  |  |  |  |  |
| To give something back |  |  |  |  |  |
| Because I felt obliged to volunteer |  |  |  |  |  |
| To be at parkrun even though I could not/did not want to walk or run (e.g. due to ill health, injury or a recovery day) |  |  |  |  |  |
| To be active in a non-judgemental environment |  |  |  |  |  |
| To be active in a physically safe environment |  |  |  |  |  |
| To improve my confidence |  |  |  |  |  |
| To contribute to my fitness |  |  |  |  |  |
| To apply or improve my skills (e.g. team work) |  |  |  |  |  |
| To have some time to myself |  |  |  |  |  |
| To be active |  |  |  |  |  |
| To be outdoors |  |  |  |  |  |
| To meet new people |  |  |  |  |  |
| To have fun |  |  |  |  |  |
| To manage my health condition, disability or illness |  |  |  |  |  |
| Other [please specify] |  |  |  |  |  |

- If participants chose Runner or walker only or Runner or walker and volunteer

Thinking about the impact of parkrun, to what extent has running or walking at parkrun changed:

|  | Much worse | Worse | No impact | Better | Much  better |
| --- | --- | --- | --- | --- | --- |
| Your physical health |  |  |  |  |  |
| Your mental wellbeing |  |  |  |  |  |
| Your life satisfaction |  |  |  |  |  |
| Your sense of personal achievement |  |  |  |  |  |
| Your happiness |  |  |  |  |  |
| Your ability to be active in a non-judgemental environment |  |  |  |  |  |
| Your ability to be active in a physically safe environment |  |  |  |  |  |
| Your fitness |  |  |  |  |  |
| The amount of time you spend with other people (e.g. family, friends or colleagues) |  |  |  |  |  |
| How much you feel part of a community |  |  |  |  |  |
| Your ability to manage your weight |  |  |  |  |  |
| Your confidence |  |  |  |  |  |
| Your opportunity to compete against others |  |  |  |  |  |
| Your opportunity to compete against yourself |  |  |  |  |  |
| The time you have to yourself |  |  |  |  |  |
| How active you are |  |  |  |  |  |
| The amount of time you spend outdoors |  |  |  |  |  |
| The number of new people you meet |  |  |  |  |  |
| Your opportunity to have fun |  |  |  |  |  |

- If participants chose Volunteer only or Runner or walker and volunteer

Thinking about the impact of parkrun, to what extent has volunteering at parkrun changed:

|  | Much worse | Worse | No impact | Better | Much  better |
| --- | --- | --- | --- | --- | --- |
| Your physical health |  |  |  |  |  |
| Your mental wellbeing |  |  |  |  |  |
| Your life satisfaction |  |  |  |  |  |
| Your sense of personal achievement |  |  |  |  |  |
| Your happiness |  |  |  |  |  |
| How much you feel part of a community |  |  |  |  |  |
| The amount of time you spend with other people (e.g. family, friends or colleagues) |  |  |  |  |  |
| Your opportunity to give something back |  |  |  |  |  |
| Your sense of obligation to volunteer |  |  |  |  |  |
| Your opportunity to be at part of parkrun eve when you are not running/walking |  |  |  |  |  |
| Your ability to be active in a non-judgemental environment |  |  |  |  |  |
| Your ability to be active in a physically safe environment |  |  |  |  |  |
| Your confidence |  |  |  |  |  |
| Your fitness |  |  |  |  |  |
| Your skills |  |  |  |  |  |
| The time you have to yourself |  |  |  |  |  |
| How active you are |  |  |  |  |  |
| The amount of time you spend outdoors |  |  |  |  |  |
| The number of new people you meet |  |  |  |  |  |
| Your opportunity to have fun |  |  |  |  |  |

If there is anything else you would like to mention about the impact of parkrun on your health and wellbeing, please write your comments here. [empty text box]
