# Supplementary material for: A generic model of life satisfaction: The case study of parkrun
Source: PLOS Glob Public Health. 2025 Oct 2;5(10):e0005065. doi: 10.1371/journal.pgph.0005065 (PMC12490765; doi:10.1371/journal.pgph.0005065)
Supplement: S2 Text — (DOCX) [file pgph.0005065.s006.docx]

# File S2 Text: reliability of survey questions.

| Cronbach's Alpha | Cronbach's Alpha Based on Standardized Items | N of Items |  |  |
| --- | --- | --- | --- | --- |
| 0.944 | 0.951 | 41 |  |  |
| Item-Total Statistics | |  |  |  |
|  | Scale Mean if Item Deleted | Scale Variance if Item Deleted | Corrected Item-Total Correlation | Cronbach's Alpha if Item Deleted |
| Life satisfaction ONS | 152.351681 | 258.087 | 0.12 | 0.951 |
| Health status code | 156.688279 | 264.507 | 0.053 | 0.946 |
| Your physical health | 155.871932 | 253.497 | 0.597 | 0.942 |
| Your mental wellbeing | 155.928069 | 251.918 | 0.645 | 0.942 |
| Your life satisfaction | 155.927522 | 251.997 | 0.681 | 0.942 |
| Your sense of personal achievement | 155.710238 | 253.428 | 0.597 | 0.942 |
| Your happiness | 155.896076 | 251.958 | 0.685 | 0.941 |
| Your ability to be active in a non-judgemental environment | 156.156297 | 250.654 | 0.583 | 0.942 |
| Your ability to be active in a physically safe environment | 156.148794 | 250.923 | 0.591 | 0.942 |
| Your fitness | 155.805629 | 254.435 | 0.554 | 0.942 |
| The amount of time you spend with other people (e.g. family, friends or colleagues) | 156.187714 | 253.699 | 0.505 | 0.943 |
| How much you feel part of a community | 155.86584 | 252.21 | 0.61 | 0.942 |
| Your ability to manage your weight | 156.439008 | 255.766 | 0.427 | 0.943 |
| Your confidence | 156.266804 | 250.988 | 0.629 | 0.942 |
| Your opportunity to compete against others | 156.253894 | 258.642 | 0.262 | 0.945 |
| Your opportunity to compete against myself | 155.810363 | 256.315 | 0.387 | 0.944 |
| The time you have to yourself | 156.574284 | 254.226 | 0.473 | 0.943 |
| How active you are | 155.909919 | 253.051 | 0.583 | 0.942 |
| The amount of time you spend outdoors | 155.989726 | 252.823 | 0.606 | 0.942 |
| The number of new people you meet | 156.088675 | 253.344 | 0.544 | 0.942 |
| Your opportunity to have fun | 155.978844 | 251.689 | 0.657 | 0.942 |
| Your physical health | 156.682468 | 254.656 | 0.563 | 0.942 |
| Your mental wellbeing | 156.213484 | 250.767 | 0.684 | 0.941 |
| Your life satisfaction | 156.125381 | 251.58 | 0.693 | 0.941 |
| Your sense of personal achievement | 156.096767 | 251.4 | 0.664 | 0.942 |
| Your Happiness | 156.151273 | 251.115 | 0.711 | 0.941 |
| How much you feel part of a community | 155.873287 | 252.097 | 0.637 | 0.942 |
| The amount of time you spend with other people (e.g. family, friends or colleagues) | 156.356762 | 253.545 | 0.54 | 0.942 |
| Your opportunity to give something back | 155.570264 | 255.156 | 0.53 | 0.943 |
| Your sense of obligation to volunteer | 156.417443 | 259.112 | 0.278 | 0.944 |
| Your opportunity to be at parkrun even when you are not running/walking | 155.821298 | 253.785 | 0.514 | 0.943 |
| Your ability to be active in a non-judgmental environment | 156.587679 | 252.582 | 0.604 | 0.942 |
| Your ability to be active in a physically safe environment | 156.653492 | 253.397 | 0.597 | 0.942 |
| Your confidence | 156.413064 | 251.568 | 0.648 | 0.942 |
| Your fitness | 156.750237 | 255.968 | 0.516 | 0.943 |
| Your skills | 156.505321 | 253.335 | 0.595 | 0.942 |
| The time you have to yourself | 156.790059 | 255.58 | 0.491 | 0.943 |
| How active you are | 156.659184 | 254.288 | 0.562 | 0.942 |
| The amount of time you spend outdoors | 156.221803 | 252.511 | 0.628 | 0.942 |
| The number of new people you meet | 156.079305 | 253.157 | 0.601 | 0.942 |
| Your opportunity to have fun | 156.258431 | 251.119 | 0.673 | 0.941 |
