# Supplementary material for: A generic model of life satisfaction: The case study of parkrun
Source: PLOS Glob Public Health. 2025 Oct 2;5(10):e0005065. doi: 10.1371/journal.pgph.0005065 (PMC12490765; doi:10.1371/journal.pgph.0005065)
Supplement: S3 Text — (DOCX) [file pgph.0005065.s007.docx]

# File S3 Text: models using previous parkrun surveys.

Table A. Multiple stepwise non-linear regression model of life satisfaction following participation in parkrun as a runner, walker or volunteer a 2018 cross-sectional survey [13]. Dependent variable: life satisfaction. Independent variables: EQ-5D-VAS (as a quadratic), age (as a quadratic in years), time registered (as a quadratic in years), gender, index of multiple deprivation, activity at registration (in categories), activity change (in categories), number of parkruns completed, number of volunteering occasions. Data is unweighted.

|  | Unstandardised Coefficients | | Standardised  Coefficients | |  |  |  |  |  |  |  |  |
| --- | --- | --- | --- | --- | --- | --- | --- | --- | --- | --- | --- | --- |
| Factor selected by model | B | Standard error | Beta | t | Significance | R | R^2^ | R^2^adj | R^2^ change | R^2^ change (% of R^2^) | Effect^†^ |  |
| (Constant) | 2.882 | 0.148 |  | 19.46 | <0.001 |  |  |  |  |  |  |  |
| EQ-5D VAS score | 0.0667 | 0.004 | 0.581 | 18.68 | <0.001 | 0.457 | 0.209 | 0.209 | 0.20877 |  |  |  |
| Age at registration squared (years) | 0.00040 | 0.000 | 0.327 | 12.86 | <0.001 | 0.477 | 0.227 | 0.227 | 0.01843 | 8.1% | Very large | |
| Age at registration | -0.0214 | 0.003 | -0.193 | -7.58 | <0.001 | 0.478 | 0.228 | 0.228 | 0.00095 | 0.4% | Very large | |
| Time registered (years) | 0.0141 | 0.004 | 0.02 | 3.58 | <0.001 | 0.479 | 0.229 | 0.229 | 0.00093 | 0.4% | Small | |
| Activity change (categories) | 0.0643 | 0.007 | 0.052 | 9.34 | <0.001 | 0.479 | 0.230 | 0.230 | 0.00068 | 0.3% | Small | |
| Activity at registration (categories) | 0.0578 | 0.007 | 0.046 | 8.20 | <0.001 | 0.481 | 0.231 | 0.231 | 0.00124 | 0.5% | Small | |
| EQ-5D VAS squared | -0.00012 | 0.000 | -0.153 | -4.93 | <0.001 | 0.481 | 0.231 | 0.231 | 0.00044 | 0.2% | Moderate | |
| Gender code (male=0, female=1) | 0.0493 | 0.013 | 0.017 | 3.80 | <0.001 | 0.481 | 0.232 | 0.232 | 0.00026 | 0.1% | Small | |
| Index of multiple deprivation* (quartile) | 0.0229 | 0.006 | 0.016 | 3.56 | <0.001 | 0.482 | 0.232 | 0.232 | 0.00025 | 0.1% | Small | |
| Runs or walks (total) | 0.000383 | 0.000 | 0.013 | 2.43 | 0.015 | 0.482 | 0.232 | 0.232 | 0.00012 | 0.05% | Small | |
| ANOVA | Sum of squares | df | Mean square | F | Significance |  |  |  |  |  |  |  |
| Regression | 18,695 | 10 | 1,870 | 1,188 | <0.001 |  |  |  |  |  |  |  |
| Residual | 61,872 | 39,329 | 1.573 |  |  |  |  |  |  |  |  |  |
| Total | 80,568 | 39,339 |  |  |  |  |  |  |  |  |  |  |

^†^ Changes in R^2^: very small<0.1%; 0.1 to 0.5% small; 0.5% to 1% moderate; 1% to 5% large; >5% very large [5].

*Quartiles 1 to 4 coded 2 to 5 in the original data

Table B. Multiple stepwise non-linear regression model of life satisfaction **change** following participation in parkrun as a runner, walker or volunteer using the previous longitudinal study [18]. Data is weighted to the full parkrun population. Dependent variable: life satisfaction change. Independent variables: EQ-5D-VAS change (as a quadratic), SWEMWBS change, age (as a quadratic), gender, index of multiple deprivation, activity at registration, activity change, number of parkruns completed as a runner/walker or as a volunteer.

|  | Unstandardised Coefficients | | Standardised  Coefficients | |  |  |  |  |  |  |
| --- | --- | --- | --- | --- | --- | --- | --- | --- | --- | --- |
| Factor selected by model | B | Standard error | Beta | t | Significance | R | R^2^ | R^2^adj | R^2^ change | Effect^†^ |
| Constant | 1.885 | 0.5388 |  | 3.498 | 0.001 |  |  |  |  |  |
| EQ-5D VAS change | 0.0299 | 0.0046 | 0.275 | 6.456 | <0.001 | 0.3659 | 0.1339 | 0.132 | 0.3659 | Very large |
| SWEMWBS change | 0.107 | 0.0167 | 0.282 | 6.408 | <0.001 | 0.4501 | 0.2026 | 0.199 | 0.1339 (33.9%) | Very large |
| Age at survey | -0.0827 | 0.0261 | -0.815 | -3.167 | 0.002 | 0.4808 | 0.2312 | 0.226 | 0.0687 (12.4%) | Very large |
| Age at survey squared | 0.000805 | 0.0003 | 0.677 | 2.649 | 0.008 | 0.4973 | 0.2473 | 0.240 | 0.0286 (6.5%) | Very large |
| Gender | 0.314 | 0.1110 | 0.122 | 2.831 | 0.005 | 0.5100 | 0.2601 | 0.251 | 0.0161 (4.9%) | Large |
| Activity change | 0.103 | 0.0403 | 0.106 | 2.543 | 0.011 | 0.5192 | 0.2696 | 0.259 | 0.0128 (3.5%) | Large |
| EQ-5D VAS change squared | -0.000423 | 0.0002 | -0.093 | -2.221 | 0.027 | 0.5273 | 0.2781 | 0.266 | 0.0095 (3.0%) | Large |
| ANOVA | Sum of squares | df | Mean square | F | Significance |  |  |  |  |  |
| Regression | 197 | 7 | 28.123 | 23.142 | <0.001 |  |  |  |  |  |
| Residual | 511 | 421 | 1.215 |  |  |  |  |  |  |  |
| Total | 708 | 428 |  |  |  |  |  |  |  |  |

^†^ Changes in R^2^: very small<0.1%; 0.1 to 0.5% small; 0.5% to 1% moderate; 1% to 5% large; >5% very large [5].
