# Supplementary material for: A generic model of life satisfaction: The case study of parkrun
Source: PLOS Glob Public Health. 2025 Oct 2;5(10):e0005065. doi: 10.1371/journal.pgph.0005065 (PMC12490765; doi:10.1371/journal.pgph.0005065)
Supplement: Attachment — Submitted filename: Haake et al 2025 Covering letter revisions.docx [file pgph.0005065.s009.docx]

6^th^ June 2025

Dear Reviewers,

**Re: A generic model of life satisfaction: the case study of parkrun**

Thank you to the reviewers, the paper has been much improved following our responses to your comments. We have tried to signpost below where are changes are using line numbers, with changes shown in the marked version of the manuscript.

**Reviewer #1:**

1. *Please provide reliability tests (i.e. Cronbach alpha scores) of all research instruments (questionnaires) used in this study to obtain data (Life Satisfaction, health status etc.). It will add required credibility to this study. Please critically discuss the reliability results of these instruments and provide relevant justification for using them as opposed to other research instruments.*

Thank you for the suggestion: we agree. The following was added to the methods in the Statistics section (lines 244 to 246):

*“Cronbach’s Alpha was used to assess the reliability of the self-reported items used in the survey, i.e., life satisfaction, health status, activity at registration and at the survey, and impact following running/walking and volunteering.”*

The following was added to the results section (lines 288 to 292) and output files added to file S4 and an additional reference [30]:

### *“Reliability of survey items*

*The reliability of the survey items used in the analysis described here were assessed using Cronbach’s Alpha. The reliability of the scores was found to be 0.935, with a minimum of 0.932 and maximum of 0.941 (see file S4). These values were in the range suggested for applied research of 0.8 or more [30].”*

The following was added to the discussion (lines 608 to 619) and two references added [35, 36]:

*“There are a number of strengths to this study. The first is that the principal outcome measure of life satisfaction has been extensively researched by the UK Statistics Authority and has been designated as an accredited official statistic [35]. Likewise, the health status question has been widely used worldwide [36] and a meta-analysis has shown that the ratings correlate inversely with mortality [37].*

*The Cronbach values for the sample using the survey described here was high at 0.93, greater than the 0.8 cutoff suggested for applied research [30]. This may even be considered too high, suggesting that the questions might have been overly repetitive in asking about subjective wellbeing. While other scales could have been used to measure wellbeing, the life satisfaction is used by governments across many countries worldwide [14] as is the health status question [35]. This makes the approach described here a methodology that might be used in other countries.”*

--

**Reviewer #2: A few areas require revision for greater clarity and impact.**

1. *First, the authors must explain the rationale for applying WELLBY valuations based on 2019 and why adjustments for 2024 economic conditions are unnecessary.*

We have revisited this issue and agree with the reviewer’s implication that the WELLBY valuation ought to be updated to 2024 prices i.e., for the year of the survey. The results section describes this and the Treasury calculations required are shown in a new version of File S3. Thus, the WELLBY value at 2024 prices was £15,935 with lower and upper limits of £12,257 and £19,612. Table 4 has been amended accordingly as has the text in the results, discussion, conclusion and abstract.

2. *Second, while the authors recognize the limitations inherent in cross-sectional data, more discussion on assumed selection biases and whether and how they might affect additionality estimates would enhance the discussion.*

Our first response to this comment was to do a sensitivity analysis on additionality shown in Table 5: a discussion was added to the results section (lines 525 to 532) as follows:

*“Sensitivity analysis of additionality and health status*

*A sensitivity analysis of additionality and health status change was carried out (Table 5), varying them both between 0 and 100% of the values in Table 4 and comparing the central-value benefit-cost ratios to the value of 5.9:1 found in population studies [31]. With no health status change, i.e., 0%, the benefit-cost ratio was greater than 5.9 if additionality was at least 40% of the original estimate, i.e., 0.181. If additionality was 10% of its original value (i.e., 0.0453), and health status did not change, the benefit cost ratio was 1.6; this increased to 5.3 as health status increased to the full proportion of the 3% estimate of change.”*

We also added discussion on biases in a new Weaknesses section of the discussion (lines 746 to 754) as follows:

*“Secondly, data was from self-report questionnaires and was likely to contain selection and adherence bias [36]. Since the participants in the study were self-selecting, they represent those who are happy to answer surveys and may represent those with a positive view of parkrun. The weighted mean life satisfaction of the sample was 7.54 while the unweighted value was 7.69 suggests that in terms of demographics at least, the sample represented those with higher levels of life satisfaction at the survey. In terms of adherence, the survey does not represent those who dropped out of participation parkrun or never participated following registration and, again, is likely to represent those with positive views of parkrun.*

*In terms of the logic model, the self-reported impacts are likely to be overly positive in magnitude. However, given that the regressions in Tables 1 and 2 are relational, the ranking may not change if those with more negative views of parkrun were included in a future survey. It is hypothesised that the ranking of the variables affecting life satisfaction would remain the same even with a less biased sample. Ideally, future research should attempt to find either a matched control group, and perhaps do surveys in-person on those unlikely to fill out online surveys or who have stopped participating in parkrun.”*

3. *Third, while parkrun activity is shown not to be significantly related to the frequency of life satisfaction (number of events), the discrepancy between this finding and reported impact (self-reported improvement) must be explored further—possibly accounting for social desirability bias or recall effects.*

This comment was the hardest for us to address and forced us to do a significant amount of data analysis to understand what was going on. This showed us that there was a confounding interaction between (1) age at the survey and (2) the number of parkruns completed. This occurred because participants who had been registered longer did more parkruns and were therefore older (since parkrun happens only once per week). Thus, we represented age at the survey as two separate variables: (1) age at registration and (2) time registered. When we did this, both age at registration and time registered were significant as expected, but the number of parkruns completed also became a significant variable in the regressions. We repeated the regression analysis for the 2018 study and found the same.

The regression model accounted for both time registered and the number of parkruns completed. The consequence for parkrun is that the number of runs or walks is positively associated with life satisfaction. The effect appears very small at 0.000266 per parkrun and equates in WELLBYs to £1.92 per parkrun per person, but when the full population is taken into account it amounts to benefits of £75m.

There are quite a number of changes to the text at the following locations and identified in the marked version of the manuscript:

- Abstract: lines 30 to 36
- Results: lines 339 to 352, lines 437 to 484, Table 3, Table 4, Figure 3, Figure 4, Figure 5a, File S5
- Discussion: lines 534 to 537 and 578 to 605
- Conclusions: sections between lines 810 and 847

Thank you for inspiring us to do this new analysis: the paper is much better for it.

Yours faithfully,

|  |  |  |
| --- | --- | --- |
| Professor Steve Haake | Dr Andy Hext | Dr Charlotte Benkowitz |
